# The Id protein Extramacrochaetae restrains the E protein Daughterless to regulate Notch, Rap1, and Sevenless within the R7 equivalence group of the *Drosophila* eye

**DOI:** 10.1242/bio.060124

**Published:** 2024-08-20

**Authors:** Venkateswara Reddy Onteddu, Abhishek Bhattacharya, Nicholas E. Baker

**Affiliations:** ^1^ Department of Genetics, Albert Einstein College of Medicine, 1300 Morris Park Avenue, Bronx, NY 10461, USA; ^2^ Department of Ophthalmology and Visual Sciences, Albert Einstein College of Medicine, 1300 Morris Park Avenue, Bronx, NY 10461, USA; ^3^ Department of Developmental and Molecular Biology, Albert Einstein College of Medicine, 1300 Morris Park Avenue, Bronx, NY 10461, USA

**Keywords:** *Drosophila* eye, Id protein, R7 photoreceptor, Daughterless, Extramacrochaetae, Proneural gene

## Abstract

The *Drosophila* Id gene *extramacrochaetae* (*emc*) is required during *Drosophila* eye development for proper cell fate specification within the R7 equivalence group. Without *emc*, R7 cells develop like R1/6 cells, and there are delays and deficits in differentiation of non-neuronal cone cells**.** Although *emc* encodes an Inhibitor of DNA-binding (Id) protein that is known to antagonize proneural bHLH protein function, no proneural gene is known for R7 or cone cell fates. These fates are also independent of *daughterless* (*da*), which encodes the ubiquitous E protein heterodimer partner of proneural bHLH proteins. We report here that the effects of *emc* mutations disappear in the absence of *da*, and are partially mimicked by forced expression of Da dimers, indicating that *emc* normally restrains *da* from interfering with R7 and cone cell specification, as occurs in *emc* mutants. *emc*, and *da*, regulate three known contributors to R7 fate, which are Notch signaling, Rap1, and Sevenless. R7 specification is partially restored to *emc* mutant cells by mutation of *RapGap1*, confirming that Rap1 activity, in addition to Notch activity, is a critical target of *emc*. These findings exemplify how mutations of an Id protein gene can affect processes that do not require any bHLH protein, by restraining Da activity within physiological bounds.

## Teaser

**Summary:** A well-known class of transcription regulator has unexpected functions in preventing developmental errors, in this case constraining development of the *Drosophila* eye.

## INTRODUCTION

Transcription factors of the helix-loop-helix (HLH) class play important roles in specification of cell fates. Multiple proneural bHLH genes act as master regulators of neural fate specification and differentiation in metazoans, including *Drosophila* and mammals (Baker and Brown, 2018; Dennis et al., 2019; Johnson, 2020)**.** The regulation of these transcription factors helps control the timing and pattern of neural development. Proneural bHLH proteins require ubiquitous bHLH proteins called E proteins as heterodimer partners. They can also heterodimerize with Inhibitor of DNA binding (Id) proteins, HLH proteins that lack basic sequences, preventing DNA binding by proneural proteins and hence preventing transcription factor function. Accordingly, E proteins and Id proteins are believed to define competence for neuronal specification and differentiation in response to proneural proteins (Bertrand et al., 2002; Ling et al., 2014; Oproescu et al., 2021; Roschger and Cabrele, 2017; Troost et al., 2015; Wang and Baker, 2015a).

**Fig. 1.**
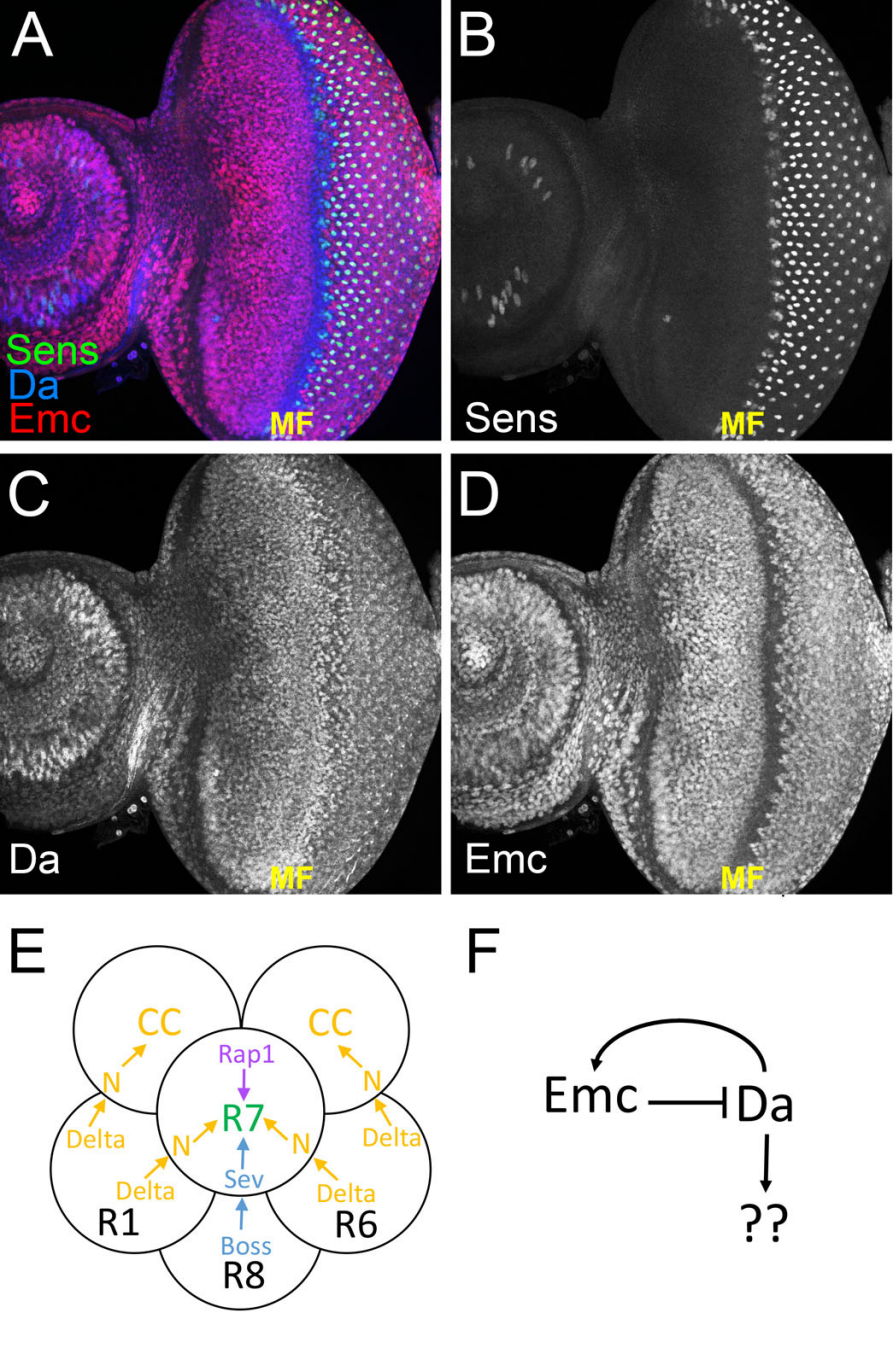
**The R7 equivalence group and HLH proteins.** (A) Eye imaginal disc showing the progression of differentiation as revealed by labeling for three proteins: Senseless (green); Emc (red) and Da (blue). Differentiation begins posterior to the morphogenetic furrow (MF). *N*>50. (B) The R8 cell of each ommatidium is labeled with anti-Senseless. Senseless expression begins within the morphogenetic furrow in groups of cells, rapidly resolving to single R8 cells. (C) Da protein is uniformly expressed except where it is increased in the morphogenetic furrow. (D) Emc protein is uniformly expressed except where it is reduced in the morphogenetic furrow. (E) The cartoon shows a subset of the R7 equivalence group and summarizes the cell–cell signals that define cell fate. The Delta protein (Dl), expressed on the surface of R1, R6 precursors, activates Notch signaling in cells that become R7 or cone cells (for simplicity, two of the four cone cells in each ommatidium are not shown here). The R7 precursor is distinguished by signaling through the receptor tyrosine kinase Sevenless (Sev), since this is the only cell in the equivalence group to contact the R8 precursor that expresses its ligand, Bride of Sevenless (Boss). Recent studies indicate multiple successive roles of N signaling in R7, which is not indicated here. Previous studies show that *emc* is required for proper R7 and cone cell differentiation, acting downstream or in parallel to Notch signaling in these cells. Among these cells, only specification of the R8 cell requires *da*. R7 fate specification also requires Rap1 activity. (F) Cartoon of the regulatory network connecting Emc and Da proteins. In the absence of competing proneural bHLH protein expression, Da is thought to be held in inactive heterodimers with Emc. In addition to restraining Da function, Emc also restrains Da expression, probably both at the levels of gene transcription and of protein stability. Da, meanwhile, maintains Emc protein levels, both through protein stabilization and potentially through gene transcription. When Emc expression is suppressed, Da levels can rise, Da homodimers form, and novel target genes become expressed, as exemplified by the transcription of *expanded* in *emc* mutant clones.

Both the proneural bHLH genes of the Achaete-Scute gene Complex (AS-C), and the unlinked proneural gene *atonal* (*ato*), are important during *Drosophila* eye development (Cadigan et al., 2002; Jarman et al., 1994). Each *Drosophila* compound eye is composed of ∼800 individual facets, called ommatidia, which consist of eight photoreceptor neurons (R1-R8), four cone cells and some other accessory cells. Cell fate specification begins during the third larval instar with R8 photoreceptor cell in the morphogenetic furrow, a visible indentation that progresses anteriorly across the eye imaginal disc until the whole eye field is differentiating (Fig. 1A,B) (Ready et al., 1976). R8 specification depends upon the proneural gene *ato* (Jarman et al., 1994). After R8 has been specified, short range signals from R8 cells recruit photoreceptor cells R2, 3,4,5. Further signals from the resulting five-cell preclusters then specify R1/6/7 cells, and finally non-neuronal cone cells in a still further round of recruitment (Freeman, 1997; Tomlinson et al., 1987; Treisman, 2013). After pupariation, sensory neural structures called inter-ommatidial bristles form in between ommatidia (Cagan and Ready, 1989; Ready et al., 1976). Inter-ommatidial bristles depend on AS-C proneural genes (Cadigan et al., 2002).

All the Ato and AS-C proteins require Daughterless (Da), their ubiquitously expressed bHLH protein heterodimer partner, for DNA binding and transcriptional activity. Da is also required, without Ato or AS-C, for the differentiation of photoreceptor cells R2-R5, and Da protein levels are elevated within the morphogenetic furrow where R8 and R2-5 cell fates are specified (Fig. 1A-C) (Brown et al., 1996). The only Id protein in *Drosophila* is encoded by the *extramacrochaetae* gene (*emc*) and is also widely expressed, but levels are reduced within the morphogenetic furrow (Fig. 1D) (Brown et al., 1995). Loss of *emc* function in the eye accelerates morphogenetic furrow progression and overall eye differentiation (Bhattacharya and Baker, 2012; Brown et al., 1995).

R1,6,7 and cone cell specification occur independently of both Da and of *ato* or AS-C (Brown et al., 1996; Jarman et al., 1994; Jiménez and Campos-Ortega, 1987). These cells are specified from an equivalence group of precursors by cell interactions. R7 specification requires the combinatorial activity of Notch and of the receptor tyrosine kinase Sevenless (Sev) (Fig. 1E) (Cooper and Bray, 2000; Mavromatakis and Tomlinson, 2012a; Miller et al., 2009; Tomlinson and Struhl, 2001). Expression of the Notch ligand Delta (Dl) on R1 and R6 cells is thought to activate Notch in the R7 precursor, and inhibit it in R1 and R6 precursors, while expression of Bride of Sevenless (Boss) on R8 cells activates signaling by its receptor Sevenless in R7 (Cooper and Bray, 2000; Mavromatakis and Tomlinson, 2012a; Miller et al., 2009; Tomlinson and Struhl, 2001; Van Vactor et al., 1991). The other cells within the equivalence group receive Dl but not Boss, and Notch signaling leads these cells to cone cell fates (Fig. 1E) (Flores et al., 2000). In *sevenless* mutants, the presumptive R7 cell is transformed to the cone cell fate by N activity (Mavromatakis and Tomlinson, 2012a; Tomlinson and Ready, 1986). Conversely, ectopic Sev signaling converts presumptive cone cells to R7 photoreceptor fate (Dickson et al., 1992; Treisman, 2013; Van Vactor et al., 1991).

R7 cell fate also requires activity of Rap1, a small GTPase proposed to synergize with Ras (Mavromatakis and Tomlinson, 2012b,a). Rap1 maintains the apical localization of Sevenless protein, as well as expression of adherens junction proteins (Baril et al., 2014), consistent with the requirement for Rap1 in maintaining cell adhesion by undifferentiated *Drosophila* imaginal disc cells (Knox and Brown, 2002). Rap1 also remodels adherens junctions during wound healing (Rothenberg et al., 2023). In vertebrates, Rap1 is required for integrity of retinal tissue in the optic tectum (Maddala et al., 2015). Rap1 activity is promoted by Guanosine Nucleotide Exchange Factors, and inhibited by GTPase activating proteins (GAPs) that convert Rap1-GTP into Rap1-GDP (Bos et al., 2007).

Surprisingly, both R7 and cone cell fates depend on *emc*, even though neither the proneural proteins nor Da are required. Loss of *emc* almost abolishes differentiation of R7 photoreceptors, as well as reducing and delaying differentiation of cone cells (Bhattacharya and Baker, 2009). Such requirements for Id proteins by cell types that do not require E proteins or other bHLH proteins have been noted before and raise the question of whether Id proteins also act through some non-bHLH-dependent mechanism (Ling et al., 2014; Wang and Baker, 2015a; Roschger and Cabrele, 2017; Oproescu et al., 2021).

One way that *emc* can act independently of proneural bHLH genes is by regulating *da*. This turns out to be why *emc* is required for growth of undifferentiated imaginal disc cells (Alonso and García-Bellido, 1988; Bhattacharya and Baker, 2011). Da protein levels increase when *emc* is mutated, due to transcriptional autoregulation and to Da protein stability in homodimers (Bhattacharya and Baker, 2011; Li and Baker, 2019). *da*, in turn, is required for Emc expression, through transcriptional regulation and increased Emc protein stability in heterodimers (Bhattacharya and Baker, 2011; Li and Baker, 2018). It is elevated Da that gives undifferentiated cells a competitive disadvantage during growth, mimicking *emc* mutations (Bhattacharya and Baker, 2011). Transcriptional targets of Da that regulate imaginal disc growth include the Hippo pathway gene *expanded* (Wang and Baker, 2015b), and potentially the cell cycle gene *string* (Andrade-Zapata and Baonza, 2014).

Here, we investigate whether Emc also restrains Da in eye development. While we reported previously that Da over-expression had no effect on R7 or cone cell development (Bhattacharya and Baker, 2009), more recent data question the interpretation of this finding. Because the Emc protein is made in excess but only stable in heterodimers with other HLH proteins such as Da, increasing Da expression increases levels of Emc/Da heterodimers by stabilizing more Emc, and the increment in Da homodimers, if any, is likely to be small (Li and Baker, 2018). We re-examined the role of *emc* in R7 and cone cell development here. We found that Emc indeed restrained Da activity during R7 photoreceptor and cone cell specification. We found evidence that Da was responsible for reduced Notch activity within the R7 equivalence group, and that *emc* also affected Sev and Rap1, the other signals controlling R7 specification. Aspects of the phenotype were mimicked by expressing Da in tethered homodimers that cannot heterodimerize with Emc. Thus, R7 and cone cell differentiation represent further examples where proneural-independent functions of *emc* reflect Da deregulation. This may be a common explanation for how *emc*, and potentially mammalian Id genes, affect developmental processes that are independent of proneural genes.

## RESULTS

### Emc promotes R7 photoreceptor cell fate by blocking Da activity

**Fig. 2.**
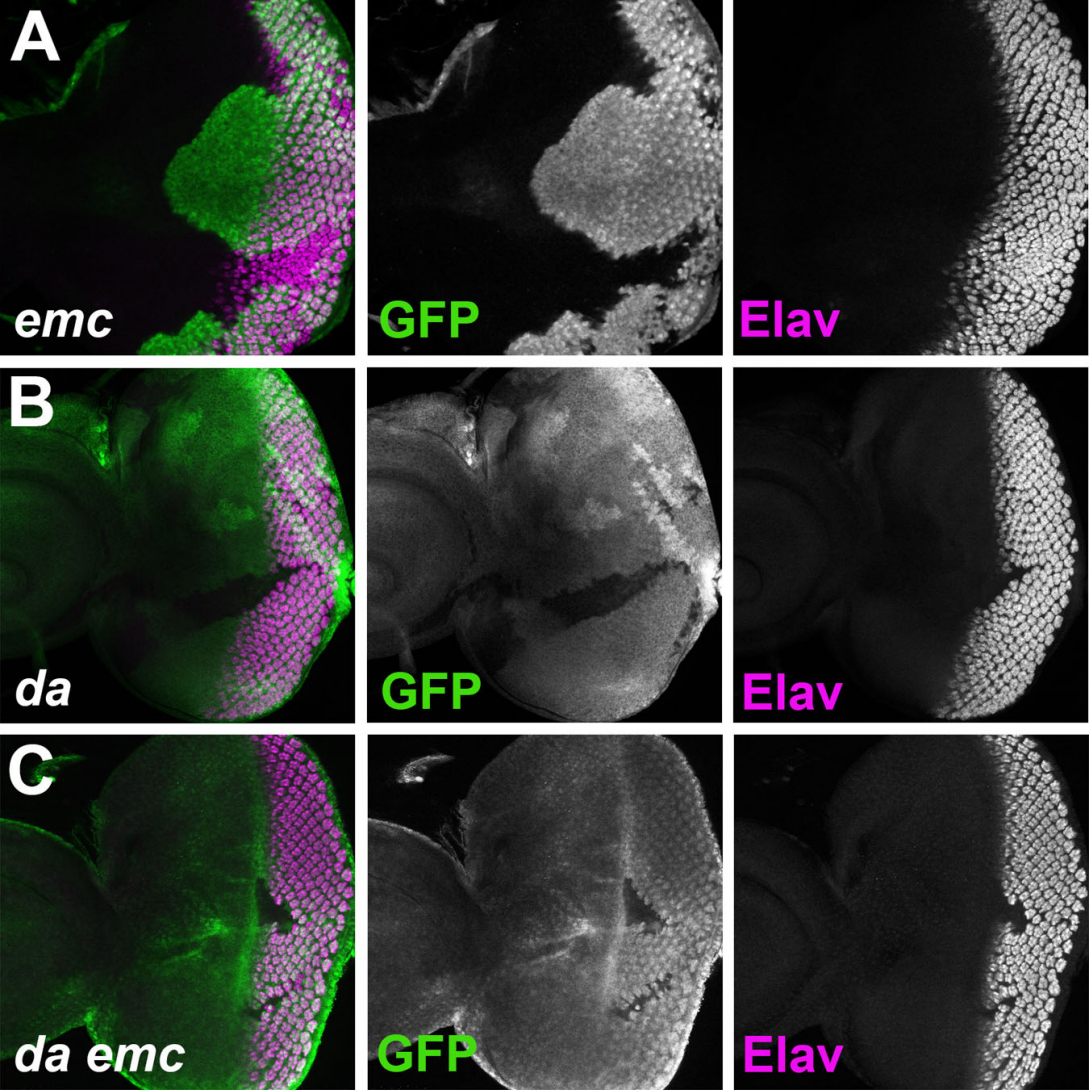
**Differentiation and survival of mutant clones.** (A) Photoreceptor differentiation occurs in *emc*-null mutant cells, although with altered timing and pattern. The mutant effects are expressed within clones in the differentiating region of the eye disc posterior to the morphogenetic furrow. Clones of *emc*-null mutant cells lack GFP labeling (green). Photoreceptor differentiation is indicated by ELAV staining (magenta). The GFP channel from panel A, showing the extent of *emc* mutant tissue posterior to the morphogenetic furrow. The Elav channel from panel A shows the differentiation in *emc* mutant clones. (B) Clones of *da* null mutant cells lack GFP labeling (green). Most differentiation is lost in *da* mutant clones (ELAV labeling in magenta). Because R8, R2, R3, R4 and R5 cells require Da cell-autonomously, photoreceptors R1, R6 and R7 differentiate only at clone borders where R8, R2, R3, R4 and R5 cells are present in neighboring non-mutant territories. The GFP channel from panel B shows that posterior eye disc regions contain only small clone remnants where all *da* mutant cells are at most a few cell diameters from wild-type territories. The ELAV channel from panel B shows that differentiation does not occur away form the boundaries of *da* clones. (C) Clones of *da emc* null mutant cells lack GFP labeling (green). Like *da* clones, photoreceptors differentiate only near clone boundaries (ELAV labeling in magenta). The GFP channel shows that posterior eye disc regions contain only small clone remnants where all *da emc* mutant cells are at most a few cell diameters from wild-type territories. The ELAV channel shows that differentiation does not occur away from the boundaries of *da emc* clones. *N*>30 for all genotypes.

To test definitively whether *emc* restrains Da activity to permit R7 specification, we compared R7 development in *da emc* double-mutant clones and *emc* single-mutant clones. If *emc* is important in R7 because it restrains Da activity, then *emc* should not be required in the absence of the *da* gene, and R7 differentiation should be restored to *da emc* double-mutant cells. It is important in this regard that previous studies show that *da* is dispensable for the development of R1, R6 and R7 cells (Brown et al., 1996), Thus, R7 cells should be able to develop in *da emc* clones if *emc* is only required to regulate *da*. If *emc* is required in R7 for other reasons, R7 cells will not differentiate in *da emc* clones. Fig. 2 shows *emc*, *da emc*, and *da* clones in eye imaginal discs from late third instars. Their comparison is complicated by their different sizes and overall differentiation status. Whereas neuronal differentiation occurs throughout *emc* clones, albeit accelerated and abnormal in pattern (Bhattacharya and Baker, 2009, 2011; Brown et al., 1995) (Fig. 2A), *da* is required cell-autonomously for the differentiation of R8, R2, R3, R4 and R5 cells (Brown et al., 1996). Because R8 and perhaps others among these cells are required to recruit R1,6,7, and cone cells, central regions of *da* clones and *da emc* clones lack ommatidial differentiation due to the combined cell-autonomous and non-autonomous effects on the various ommatidial cell types (Fig. 2B,C). Cell-autonomous roles of *emc* and *da* in R7 can still be assessed in genetic mosaics, where R8 and R2, R3, R4 and R5 cells can be wild type for *da* while other cells are not. Such mosaic ommatidia occur at the boundaries of *da* and *da emc* clones (Fig. 2B,C).

The lack of differentiation within *da* and *da emc* clones leads to a further difference from *emc* clones. Since cell survival in the eye disc posterior to the furrow depends on signals emanating from differentiating ommatidia (Baker, 2001), the centers of *da* clones lack survival signals. Only small *da* clones were found far posterior to the furrow, reflecting rescue of *da* mutant cells near the clone boundaries by survival signals coming from wild-type cells and from differentiating R1, R6 and R7 cells (Fig. 2B). The *da emc* clones shared this same phenotype; only small *da emc* clones were found posterior to the furrow (Fig. 2C).

**Fig. 3.**
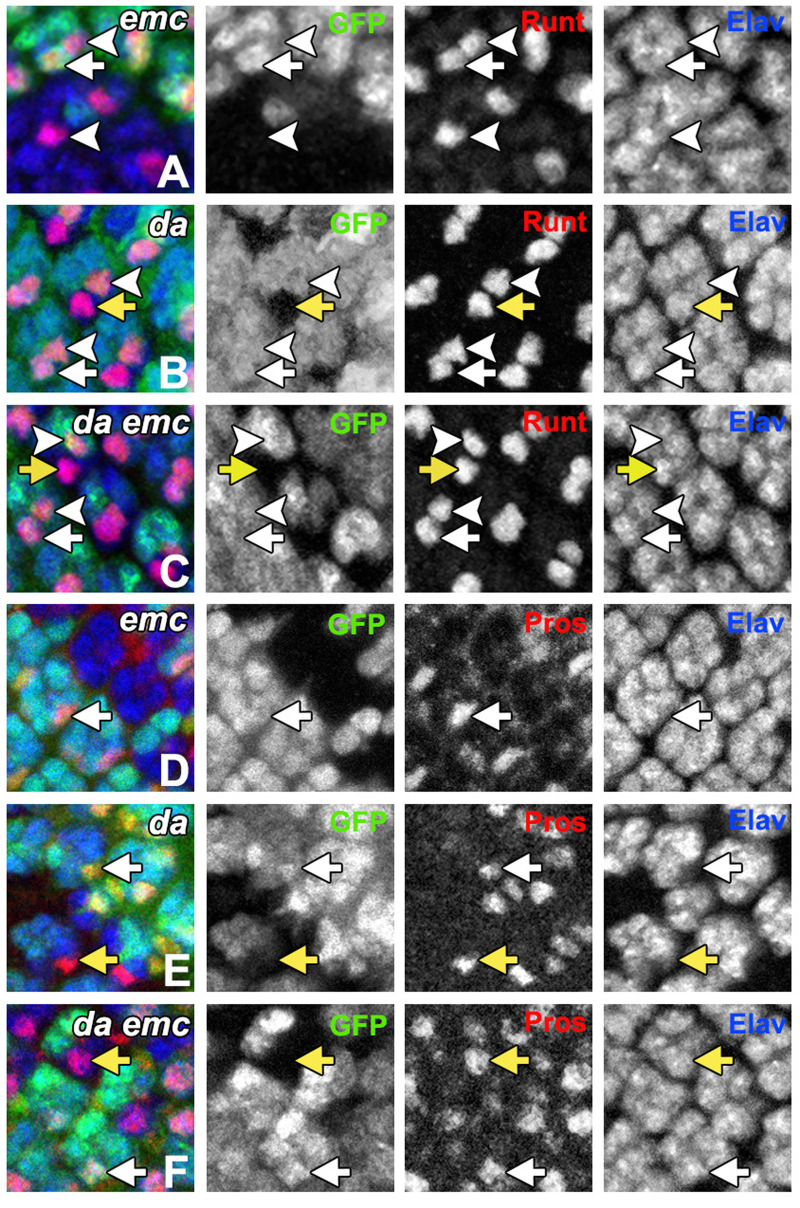
***da* is epistatic to *emc* in R7.** (A-C) Runt and Elav labeling at the boundaries of mutant clones in the posterior eye disc around columns 9-14. Whereas Elav is expressed in all photoreceptor nuclei, Runt is expressed in R7 and R8 only. R7 and R8 nuclei can be distinguished by neighbor relationships and by location in the apical-basal axis. Panels D,E,F show Prospero and Elav labeling around columns 10-14. High Pros levels are specific for R7 and cone cell precursors. Arrows indicate R7 precursors, chevrons indicate R8 precursors. Arrows and chevrons are white for genetically wild-type nuclei, yellow for mutant nuclei. Mutant cells lack GFP marker. Nuclear labels are maximum projected in the Z-axis; nuclear profiles from distinct cells may overlap in this view. (A) Only R8 cells expressed Runt inside *emc* mutant clones (chevrons). R7 precursor cells (arrows) were restricted to wild-type regions in 84/87 cases. (B) Mutant R7-like cells express Runt at the boundaries of *da* mutant clones (yellow arrows) in 40/41 cases. (C) Mutant R7-like cells express Runt at the boundaries of *da emc* mutant clones (yellow arrows) in 25/25 cases. (D) Pros is expressed in R7-like cells of wild-type territories (arrows) but not within *emc* mutant clones. *N*>25. (E) Pros is expressed in R7-like cells within *da* mutant clones (yellow arrows). *N*>25. (F) Pros is expressed in R7-like cells within *da emc* mutant clones (yellow arrows). *N*>25.

In *emc* mutant clones, cells occupying the R7 position in the ommatidium are not R7-like; they continue to express the pan-neuronal marker Elav, but fail to express at least four R7 cell markers [Runt, Prospero, Spalt, E(spl)mδ], and instead express Seven-up (Svp), a marker of R1/6 cells (Bhattacharya and Baker, 2009). Here, we used loss of Runt or Pros to follow the transformation of the R7 cell in *emc* mutants (Fig. 3). Runt is normally expressed by wild-type R7 cells from column 8 or 9 onwards, as well as by R8 cells (Kaminker et al., 2002). In the mosaic ommatidia that survive in *da* mutant clones, cells in the R7 position expressed both Elav and Runt (Fig. 3B; 40/41 cases), resembling normal R7 development. In *da emc* double mutant mosaic ommatidia that developed at the edge of *da emc* clones, cells in the R7 position also expressed Runt, which is typical of R7 cells (Fig. 3C; 25/25 cases). Therefore, *da* was epistatic to *emc* for these aspects of R7 development, indicating that elevated Da affects R7 development in *emc* mutant clones. Prospero is expressed in R7 cells from column 7 or 8 onwards (Kauffmann et al., 1996). Cells in the R7 position in *emc* mutant clones lack Pros expression (Fig. 3D) (Bhattacharya and Baker, 2009). By contrast, in both *da* and *da emc* clones, cells in the R7 position expressed both Elav and Prospero, confirming their R7-like differentiation with this second marker (40/41 for *da*, 25/25 for *da emc*; Fig. 3E,F). We can not exclude that examination of further markers, such as Svp, might reveal that cells in *da emc* clones occupy an intermediate state with some R7 and some R1/6-like properties. Taken together, however, these results do indicate that the failure of R7 development in *emc* clones depends on a cell-autonomous consequence of Da activity. There was no evidence for a requirement for *emc* in the absence of *da*.

### Emc promoted cone cell fate by modulating Da activity

**Fig. 4.**
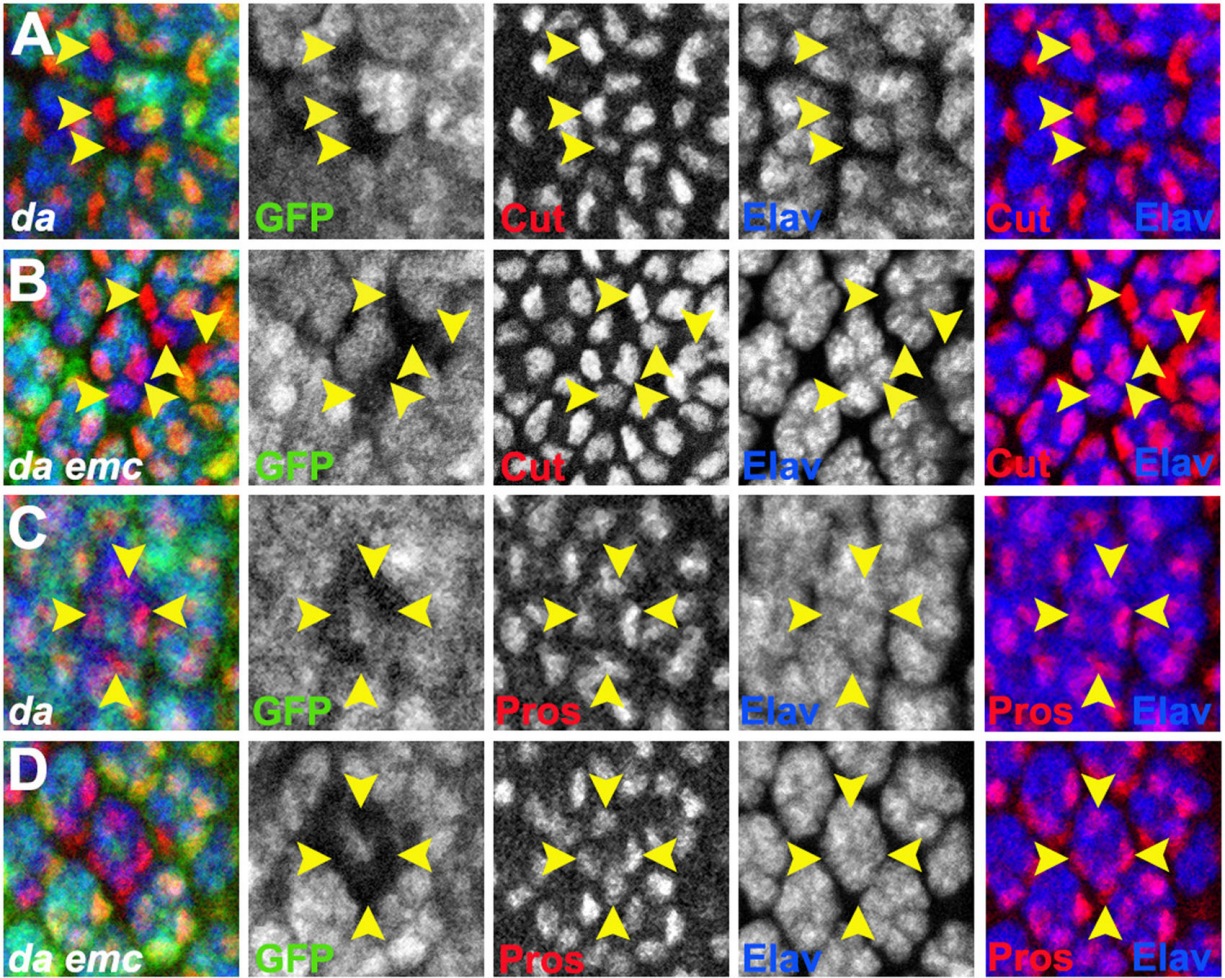
***da* is epistatic to *emc* in cone cells.** Panels show Cut or Pros expression in cone cell precursors around columns 15 and 25, respectively. Clones mutant for *da* or *da emc* lack GFP expression. Yellow chevrons indicate cone cell precursors from mutant genotypes. Nuclear labels are maximum projected in the Z-axis; nuclear profiles from distinct cells may overlap in this view. (A) Cone cell precursors express Cut in the absence of *da*. (B) Cone cell precursors express Cut in *da emc* clones. (C) Cone cell precursors express Pros in the absence of *da*. Analysis of individual confocal planes confirms that they lack Elav expression (not shown). (D) Cone cell precursors express Pros in *da emc* clones. Analysis of individual confocal planes confirms that they lack Elav expression (not shown). Number of ommatidia scored >25 for each experiment.

In addition to R7 photoreceptor fate specification, *emc* is also required for the specification of non-neuronal cone cells. Only about half as many cone cells are specified in *emc* mutant ommatidia, and cone cells that remain delay expression of the homeobox transcription factor Cut by 2 to 3 columns and of Pros by 3 to 4 columns (Bhattacharya and Baker, 2009). To test whether Emc regulates cone cell specification through Da activity, cone cell development in mosaic ommatidia simultaneously mutant for both *emc* and *da* was compared with ommatidia mosaic for either *emc* or *da* alone (Fig. 4). As for R7 differentiation, cone cells were only recovered in mosaic ommatidia at boundaries of *da* or *da emc* clones. Cone cells expressing Cut or Pros but not Elav were recovered in *da* mutant mosaic ommatidia (Fig. 4A,C). The *da emc* double mutant mosaic ommatidia developed cone cells expressing Cut or Pros at similar rates (Fig. 4B,D). Although it is difficult to compare the frequency of cone cell differentiation to that in *emc* clones because only mosaic ommatidia at clone boundaries can be recovered for the *da* and *da emc* genotypes, there was no evidence that cone cell differentiation was delayed in *da* mutant cells, or that the timing of Cut or Pros expression was delayed in *da emc* cone cells compared to *da* cone cells. These results suggested that *emc* was not required for the rate or timing of cone cell differentiation in the absence of *da*, consistent with the notion that elevated Da is the cause of cone cell differentiation defects in *emc* clones.

### Emc regulates expression of E(spl)-C genes in R7 cells through Da

**Fig. 5.**
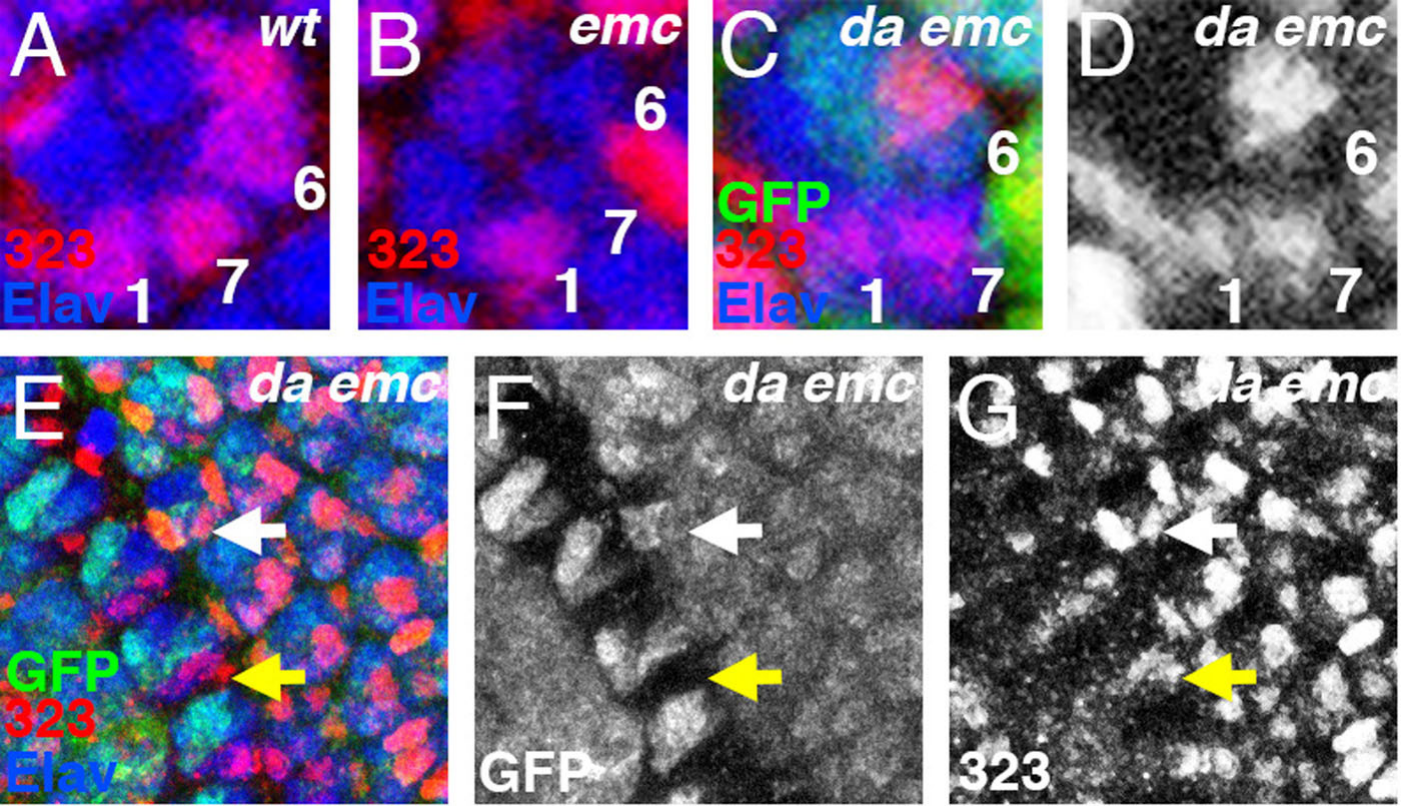
**Da delays E(spl) expression in *emc* mutants.** Panels show E(spl) proteins expressed in the R7 equivalence group, as labeled by mAb323 (red). Nuclei photoreceptor cells are marked with Elav (blue) and where relevant, clones identified by the absence of GFP (green) (A) In wild type, E(spl) proteins are detected in R1/6 precursors from column 6 and in R7 precursors from columns 8/9-15/16. (B) Within *emc* clones, E(spl) expression is delayed or absent in R1/6/7 cells. The example shows E(spl) protein detected only in R1. (C,D) In *da emc* clones, E(spl) expression is restored. The example shows expression in *da emc* mutant R1 and R7 cells from a mosaic ommatidium. Note that R6, which shows weaker expression that is above background, is not mutant in this ommatidium. (E) Lower magnification view of *da emc* mutant clone between columns 7-12. (F) GFP channel (G) E(spl) expression in mutant R7 precursor cells in column 9 (e.g. yellow arrow) occurs contemporaneously with E(spl) expression in wild-type R7 precursor cells (e.g. white arrow). 38/38 cases.

The E(spl)-C includes seven genes that encode bHLH proteins of a distinct class that are transcribed in response to Notch signaling and function as transcriptional repressors that antagonize neurogenesis (Bray, 2006; Delidakis and Artavanis-Tsakonas, 1992; Jennings et al., 1994). A monoclonal antibody that detects four of the seven E(spl) bHLH proteins detects expression in many eye disc cells posterior to the morphogenetic furrow (Baker et al., 1996; Dokucu et al., 1996; Jennings et al., 1994). All the expression in differentiating R cells depends on Notch, although this is not the case for expression in undifferentiated retinal precursor cells (Baker and Yu, 1997). Previously, we reported that E(spl)-C protein expression in the R1, R6 and R7 cells was delayed in *emc* mutants (Bhattacharya and Baker, 2009) (Fig. 5A,B). Thus, *emc* contributes to Notch signaling in R1, R6 and R7 cells. To address whether it was Da that inhibits Notch signaling in *emc* mutants, E(spl) antibody was used to label *da emc* clones. In contrast to *emc* mutant clones (Fig. 5B), E(spl) expression was restored to *da emc* mutant R1, R6 and R7 cells, and not delayed (Fig. 5C-G). This was consistent with Da being the cause of reduced Notch signaling in *emc* mutants. We showed previously that *emc* mutations affected a transcriptional reporter of Notch signaling as well as E(spl) proteins (Bhattacharya and Baker, 2009). Because we studied E(spl) protein in *da emc* clones, we cannot distinguish whether Notch signaling is restored at the level of N signal transduction to the nucleus, or by direct Da-E(spl) protein interactions and changes in E(spl) protein stability in the absence of Da (Zarifi et al., 2012) (Kiparaki et al., 2015).

### Over-expression of tethered form of Da is sufficient to cause cone cell defects

**Fig. 6.**
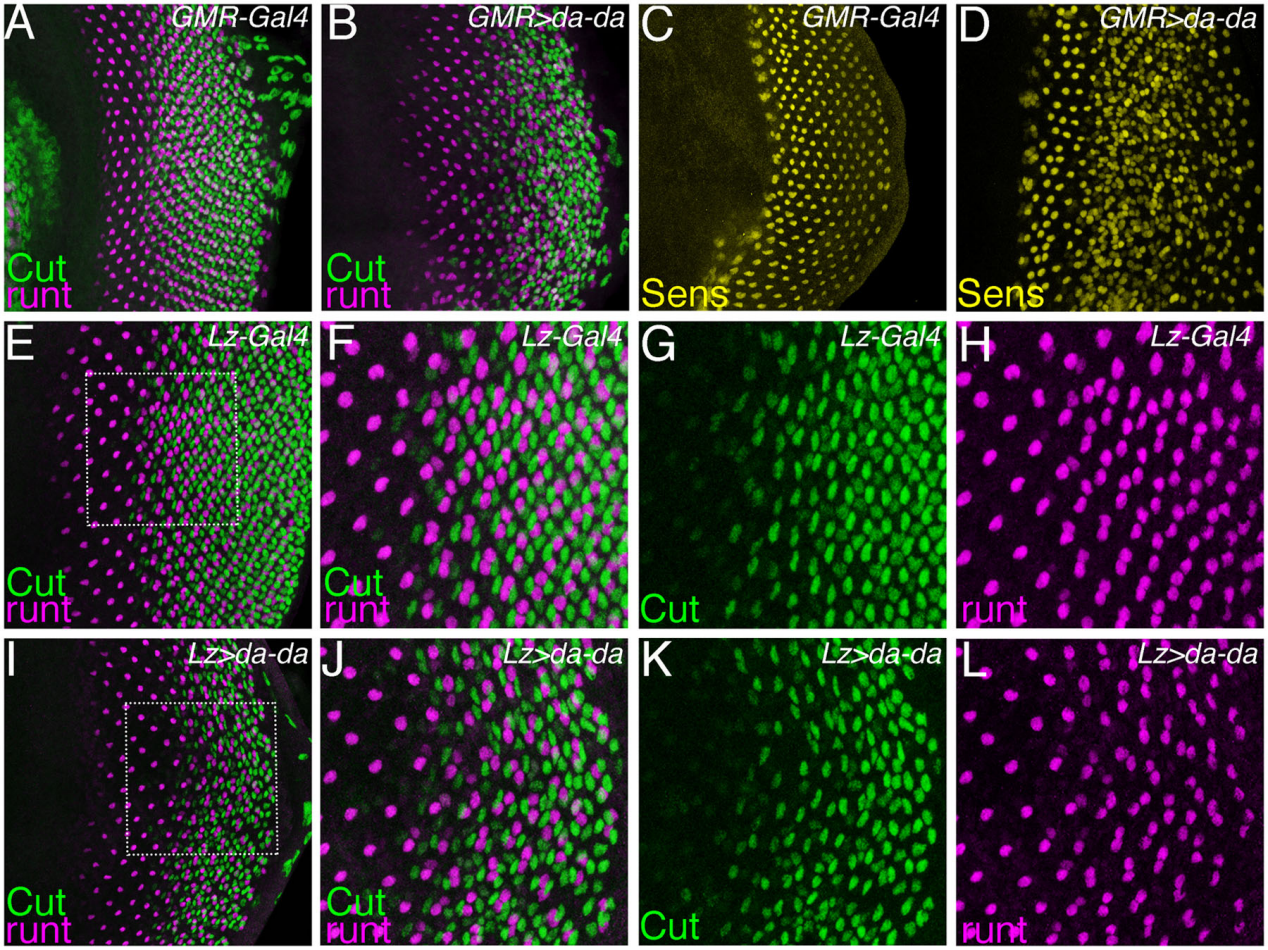
**Elevated levels of Da cause differentiation defects.** Eye primordial tissue was stained with antibodies to identify photoreceptor unit defects resulting from elevated expression of Da. (A-D) Runt in magenta. Cut in green. Senseless in yellow. (A) Control eye disc shows four-cell arrangement with cone cells placed one cell each at anterior, posterior, equatorial, and polar positions. *N*=8. (B) Forced expression of tethered Da dimer immediately posterior to the morphogenetic furrow using GMR-Gal4 caused disruption in the arrangement of cone cells. *N*=10. (C) Control eye disc shows regular arrangement of R8 cells. *N*=1 (D) Forced expression of tethered Da dimer with GMR-Gal4 increased the number of R8 cells and perturbed their regular spacing. *N*=2 (E-L) Runt labeling of R7 and R8 cells in magenta, Cut in green. (E) Control eye discs show four cone cells per ommatidium. Doublets of Runt labelling indicate the specification of R7 in addition to R8. *N*=11 (H-J) enlargement of boxed region from panel E. (I) Expression of tethered Da dimer using *lz-Gal4* perturbed cone cell arrangement with intermittent loss of cone cells. *N*=11. (J-K) enlargement of boxed region from panel I. *Genotypes*: *w; GMR Gal4/+* (B) *w; GMR Gal4/+; UAS-da-da/+* (C) *w; GMR Gal4/+* (D) *w; GMR Gal4/+; UAS-da-da/+* (E-H) *yw, lz-Gal4/+* (I-L) *yw, lz-Gal4/+; +; UAS-da-da/+*.

It was surprising that *da* was required for R7 and cone cell fates, because we previously reported that *da* overexpression had no effect on these cell types. More recent studies make it clear that Emc protein is synthesized in excess and stabilized in Da heterodimers (Li and Baker, 2018). Accordingly, elevating Da expression also elevates Emc protein levels, primarily increasing levels of inactive Da/Emc heterodimer, potentially without little effect on Da activity. A better route to elevate Da activity is to express tethered Da dimers unable to heterodimerize with Emc (Castanon et al., 2001). Expressing tethered Da dimers using an actin-Gal4 transgene conditionally activated by excision of an FRT cassette (the actin flip-on method) led to tiny clones, reflecting inhibitory effects of Da on growth and survival, and effects on fate specification were difficult to assess. Therefore, we turned to expression under Gal4 control. Expression of Da dimers posterior to the furrow using GMR-Gal4 led to more cone and R7-like cells, but there was also an increase in R8 cell numbers, consistent with a role for Da in R8 specification within the morphogenetic furrow (Fig. 6A-D). Because R8 is directly or indirectly responsible for inducing many ommatidial cell fates, effects on R7 and cone cell numbers could be indirect consequences of excess R8 specification. Accordingly, we expressed da dimers using Lz-Gal4, which is active in the progenitor cell pool that remains after the five-cell R8, R2, R3, R4, R5 preclusters have formed (Crew et al., 1997). This did not affect R7 cell specification but caused disorganization of cone cells in the eye disc and frequent loss of cone cells (Fig. 6E-J) as labelled by the cone cell marker Cut. These eye disc cone cell defects manifest into a rough eye in the adult *Drosophila* (Fig. 8A,B), similar to eye defects observed by *emc* knockdown (Fig. 8C). Inter-ommatidial bristles were also abnormal. Simultaneously removing both *emc* and *da* using RNAi rescued eye defects to wild type, confirming that defects of *emc* knockdown were due to Da activity (Fig. 8D).

### Emc regulates expression of Sev and RapGap1

**Fig. 7.**
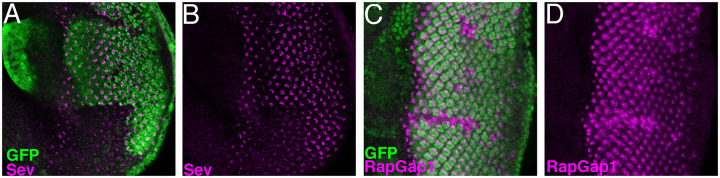
**Emc regulates expression of signaling proteins.** (A,B) Eye disc are stained for RTK- Sevenless in magenta. (A) Merged image showing Sevenless levels (magenta) inside and outside *emc* clones (lacking green GFP labeling). (B) Strong reduction of Sevenless levels in *emc* clone regions. *N*=5. (C,D) Eye disc are stained for RapGap1 in magenta (C) Merged image shows RapGap1 levels (magenta) inside and outside *emc* clone regions (lacking GFP labeling in green) (D) *emc* clone lacks GFP. (P) RapGap1 levels are strongly elevated within *emc* clones. An enlarged region of this disc is shown in Fig. S7. *N*=6 *Genotypes*: *ywhsF*; *emcAP6* FRT80/*[UbiGFP] M(3)67C* FRT80.

Having found that *emc* regulates eye development through Da, and impacts N signaling, we also checked the effects of *emc* on Sev and Rap1. We report that expression of Sev protein was cell-autonomously reduced within *emc* mutant clones. Sev is expressed in many cells, not only R7 precursors (Tomlinson et al., 1987), and this overall pattern was affected in *emc* mutant clones (Fig. 7A,B). Concomitantly, we found that expression of RapGap1 protein, a negative regulator of Rap1 activity, was cell-autonomously elevated within *emc* mutant clones. RapGap1 protein is expressed posterior to the morphogenetic furrow (Chen et al., 1997). The expression pattern was generally elevated in *emc* mutant clones (Fig. S7).

### Rap1 is modified by Da activity

**Fig. 8.**
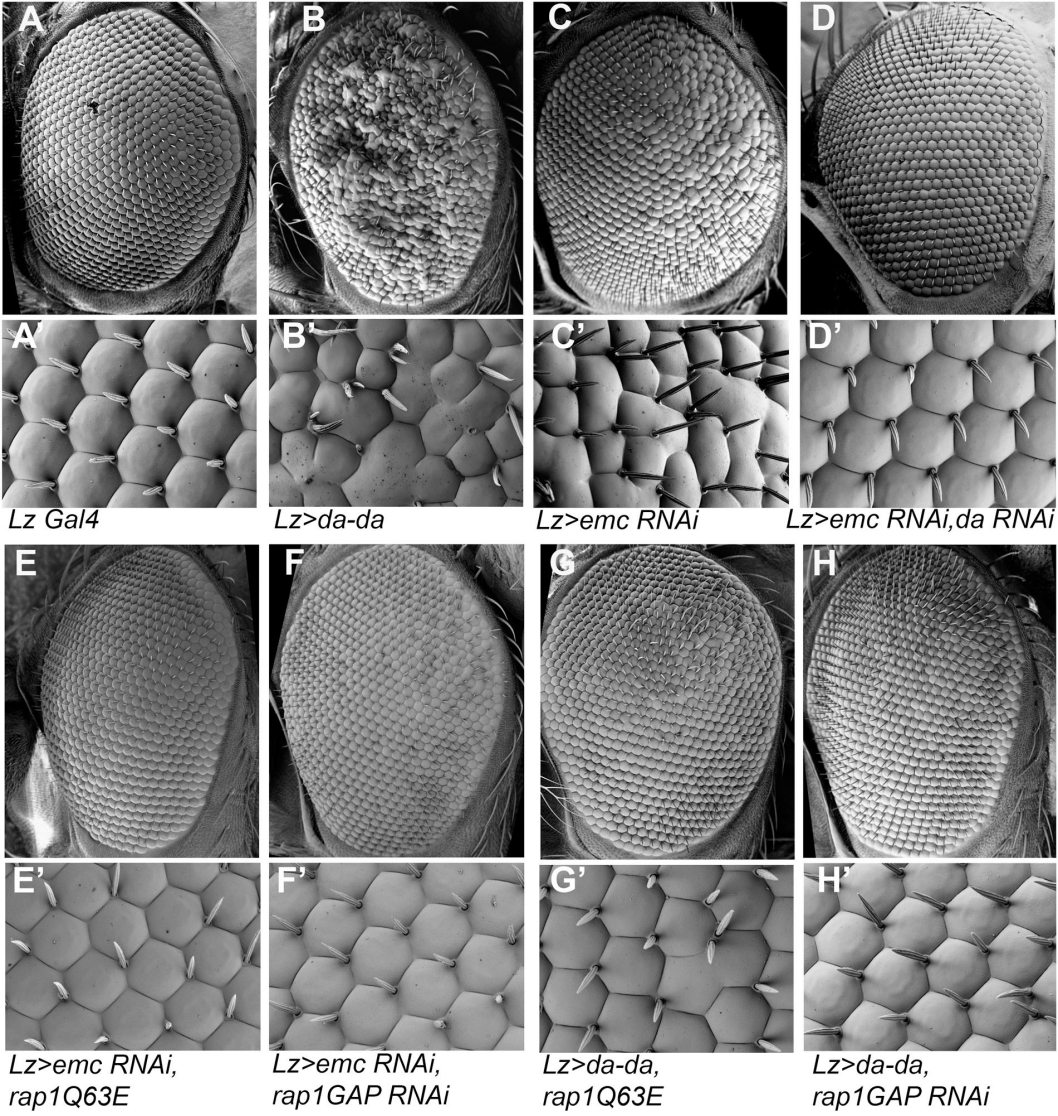
**Eye phenotypes of *emc* knockdown or forced Da expression are similar, and suppressed by Rap1 signaling.** Scanning electron microscope pictures of *Drosophila* eye morphology. Lz Gal4 was present throughout. The upper panel of an individual image display whole eye, enlarged below. *N*=3 for each genotype. (A,A′) Control (Lz-Gal4/+) condition show a regular arrangement of photoreceptors; (B,B′) Tethered Da dimer expression caused a rough eye; (C,C′) *emc* knockdown shows a rough eye; (D,D′) simultaneous knockdown of *da* rescued *emc* knockdown morphology; (E,E′) Activated form of Rap1 rescued *emc* knockdown morphology; (F,F′) simultaneous knockdown of *rapGap1* rescued *emc* knockdown morphology; (G,G′) Activated form of Rap1 rescued the morphology of eyes expressing tethered Da dimers; (H,H′) *rapGap1* knockdown rescued the morphology of eyes expressing tethered Da dimers. *Genotypes*: (A) *yw, lz-Gal4, UAS-GFP/+* (B) *yw, lz-Gal4, UAS-GFP/+; UAS- emc RNAi /+* (C) *yw, lz-Gal4, UAS-GFP/+; UAS- emc RNAi /+; UAS-da RNAi/+* (D) *yw, lz-Gal4, UAS-GFP/+; +; UAS-da-da/+* (E) *yw, lz-Gal4, UAS-GFP/+; UAS- emc RNAi /+; UASp GFP-Rap1Q63E/+* (F) *yw, lz-Gal4, UAS-GFP/+; UAS- emc RNAi /+; USA-RapGap1 RNAi /+* (G) *yw, lz-Gal4, UAS-GFP/+; +; UAS-da-da/UASp GFP-Rap1Q63E* (H) *yw, lz-Gal4, UAS-GFP/+; +; UAS-da-da/USA-RapGap1 RNAi*.

**Fig. 9.**
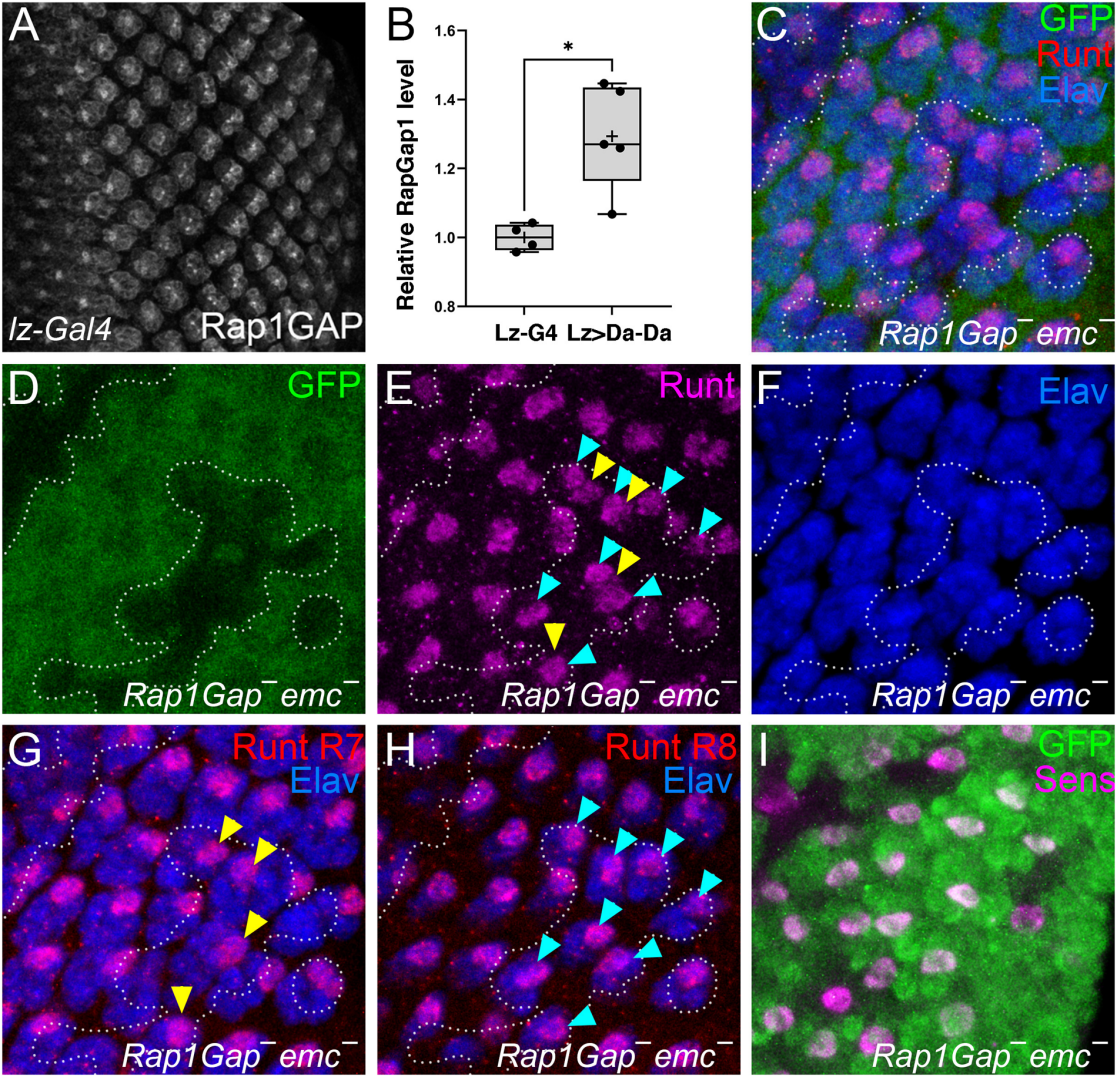
**Emc regulation of Rap1 is mediated by Da and RapGap1.** Antibody-stained eye discs are shown. (A) In control discs (Lz-Gal4/+) RapGap1 protein is detected in photoreceptor neurons developing posterior to the morphogenetic furrow. (B) RapGap1 expression was increased after Da-Da expression. Pixel intensity ratios are displayed as box/whisker plots, sample mean indicated by ‘+’ sign. The two-tailed *P*-value is 0.0159 (Mann–Whitney test). (C-I) *emc* mutant clones in the *RapGap1* mutant background Runt in magenta. Elav in blue. *N*=4. (C) Merged file showing *emc* mutant clones (lacking GFP in green) with R7/8 photoreceptors labeled for Runt in magenta and all photoreceptors labelled for Elav in blue. (D) *emc* mutant clones in the *RapGap1* mutant background labelled for the absence of GFP depicted by a dotted outline. (E) *emc* mutant clones in the *RapGap1* mutant background contained R7 cells. R8 cells indicated by a blue arrowhead, R7 cells by yellow arrowheads. (F) Elav labeling to detect photoreceptor nuclei in blue. (G) Apical optical slices were selectively merged to detect Runt-labelled R7 photoreceptors. (H) Basal optical slices were selectively merged to detect Runt labelled R8 photoreceptors. (I) Cells inside and outside *emc* mutant clones in the *RapGap1* mutant background contained single Senseless-positive R8 cells (magenta) per ommatidium. Genotypes: (A,B) *yw*, *lz-Gal4*, UAS-*GFP/+*; UAS-*da-da*/+ (C-I) *ywhsF*; *RapGap1^22^*; *emcAP6* FRT80/*[UbiGFP] M (3)67C* FRT80.

To see how altered signaling activities contributed to *emc* mutant phenotypes, we turned to genetic interactions. Expression of an activated form of Rap1 (*Rap1Q63E*) (Bonello et al., 2018) rescued the Lz-Gal4 *emc* knockdown mediated rough eye phenotype, almost to wild type (Fig. 8E,E′). RNAi knockdown of *RapGap1* resulted in a similar rescue of Lz-Gal4 *emc* knockdown (Fig. 8F,F′). Similar suppression was seen for the phenotype of Lz-Gal4 driving Da homodimer expression (Fig. 8G,G′-H,H′). Also, we found that Da overexpression was sufficient to elevate RapGap1 levels (Fig. 9A,B; Fig. S9). In the wild type, RapGap1 protein appeared in the typical R cell differentiation sequence, first in R8, R2 and R5 cells, then in R3 and R4 cells, and finally in the R1, R6 and R7 cell precursors (Fig. 9A). RapGap1 mostly seems to be expressed in R1-8 cells, little or none being detected in other eye disc cells (Fig. S9). Protein appeared mostly cytoplasmic, and prominent in R cell axons and microvilli. These results suggested that reduced Rap1 activity contributed to the *emc* phenotype. To test this definitively, *RapGap1 emc* double mutant cells were examined by inducing *emc* mutant clones in the homozygously viable *rapGap1* mutant background. If elevated RapGap1 is contributing to the failure of R7 specification in *emc* mutants, *emc* mutant clones should show more normal R7 specification in the *rapGap1* mutant background. As predicted, R7 cell specification was rescued substantially in clones of *rapGap1 emc* double mutant cells (Fig. 9C-H; 30/53 cases). No effect on R8 specification was observed (Fig. 9I). The *rapGap1* mutant background alone has no effect on R7 specification (Fig. 9E,G,H).

## DISCUSSION

The *emc* gene encodes the sole *Drosophila* representative of the Id protein family, whose members exert diverse effects on development in *Drosophila* and in mammals. The Id proteins are well known as antagonists of bHLH protein DNA binding and function, including myogenic and proneural genes as well as E proteins (Ling et al., 2014; Roschger and Cabrele, 2017; Wang and Baker, 2015b). Aspects of the *emc* phenotype in *Drosophila* affect processes that are independent of these bHLH proteins, raising the possibility of additional mechanisms. How Id proteins can function seemingly independently of its heterodimer binding partners is also a question in mammals, where four Id protein genes exist, and it is difficult to attribute all the phenotypes of individual Id gene knockouts to the many mammalian proneural bHLH genes (Ling et al., 2014; Roschger and Cabrele, 2017; Wang and Baker, 2015a).

**Fig. 10.**
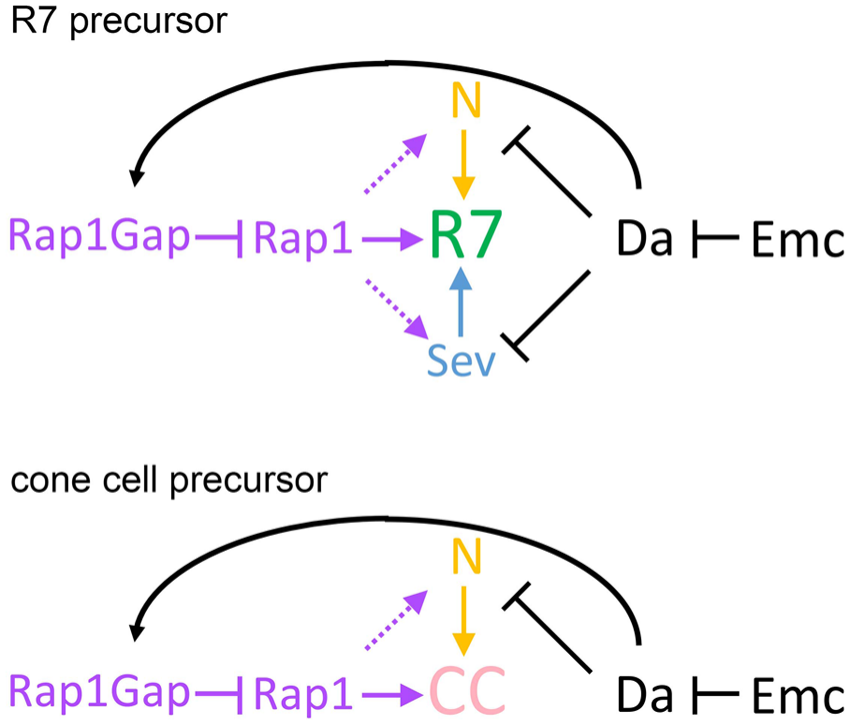
**Model for Emc function in the R7 equivalence group.** Updated cartoon view signaling within the R7 equivalence group. In the absence of *emc*, unrestrained Da activity impinges on all three pathways affecting R7 cell fate. Da inhibits Sev expression and N activity, as well as elevating expression of RapGap1, which inhibits Rap1 activity. Previous work shows that N activity, albeit at a high level through N intracellular domain, restores R7 specification to *emc* mutant clones. We additionally find that restoring Rap1 activity by removing *RapGap1* also restores most R7 specification. It is an intriguing possibility that Rap1 might act on Sev and/or N activities, through its role in maintaining contacts between adhesive cells (Baril et al., 2014) or otherwise. It is possible that the effect on Sev expression is a consequence of altered N activity (Mavromatakis and Tomlinson, 2012b). Our data indicate that Rap1 likely contributes to cone cell specification in addition to R7, based on adult eye morphology after *emc* knockdown or expression of tethered Da dimer.

We previously showed that the role of *emc* in proper growth of undifferentiated imaginal disc cells is due to its restraint of the ubiquitous E protein Da, which is thought to homodimerize in the absence of *emc* (Bhattacharya and Baker, 2011). This is even though imaginal disc growth does not depend on *da* in the wild type (Bhattacharya and Baker, 2011). A similar conclusion was reached by others (Andrade-Zapata and Baonza, 2014). Here, we report that aspects of *emc* during eye development also reflect requirements to restrain *da*, this time in post-mitotic cells, and identify some of the target pathways (Fig. 10).

Among multiple roles of *emc* during eye development are requirements to prevent R7 precursor cells from adopting R1/6 fate, and for the specification and timely differentiation of cone cells. No proneural bHLH proteins are known to be required for R7 or for cone cell development, and *da* is also not required for R7 or for cone cell fates. Here we show through analysis of *da emc* double mutant clones, and also of eyes depleted for both *emc* and *da*, that the *emc* phenotypes depend on *da*, and must be consequences of *da* activity when it is not restrained by *emc* (Fig. 10).

We had not previously observed R7 or cone cell defects after over-expressing *da* in the eye, which would be predicted to occur. This could be explained by the stabilization of Emc protein by heterodimerization with Da, through which elevated Da expression leads mostly to increased amounts of inactive Emc/Da heterodimer, and little or no increase in Da activity (Li and Baker, 2018). We now report that expression of tethered a Da-Da dimer, which is not subject to inhibition by heterodimerization with Emc, did lead to cone cell defects similar to *emc* depletion (Figs 7, 8). We do not know whether we have not observed any effect of Da-Da dimers on R7 for technical reasons, or because Da alone is not sufficient for R7 transformation to R1/6, although it is required.

We found previously that *emc* was required for the proper level and timing of N activity, which is required for both R7 and cone cell fate specification (Bhattacharya and Baker, 2009). Here, we established that N activity in the R7 equivalence group, as measured by E(spl) protein expression, was affected in *emc* mutants in a *da*-dependent manner. We also found that two other signaling pathways required for R7 specification, Sevenless and Rap1, depended on *emc*. Sevenless protein expression levels depended on *emc* function, and *emc* was required to restrain levels of RapGap1, a negative regulator of Rap1 activity. The increased RapGap1 levels, expected to diminish Rap1 function in *emc* mutant cells, were functionally significant, because expression of activated Rap1 suppressed phenotypes of *emc* depletion or tethered Da-Da expression, and mutation of *RapGap1* restored normal R7 specification to *emc* mutant clones. In addition to the absence of R7 cells from *Rap1* mutants, cone cells are disorganized and sometimes missing, resembling the Da dimer expression defects (Mavromatakis and Tomlinson, 2012b) (Fig. 10).

In undifferentiated imaginal disc cells, Emc prevents transcriptional activation of *ex*, a gene in the Hippo pathway, by Da (Wang and Baker, 2015b). Another role of *emc* has also since been attributed to unrestrained *da* activity. The *emc* gene is required for left–right asymmetrical development of the *Drosophila* hindgut, in which the potential *da* target encodes a Myosin, MyoID (Ishibashi et al., 2019). The direct transcriptional target, or targets, of *da* in the eye are not yet clear. It is interesting that restoring either Rap1 or N activity seems sufficient to restore R7 differentiation. One explanation could be that N, Sev and RapGap1 act in a pathway, for example that N activates RapGap1 expression, which is required in turn for Sev expression. It has been proposed previously that *Sev* gene expression is likely Notch-dependent, which could potentially account for the reduction in Sev expression in *emc* mutant clones where N activity is diminished (Tomlinson et al., 2011). Proper Sev protein expression also requires Rap1 activity, possibly through the maintenance of cellular adhesion structures (Baril et al., 2014). RapGap1 expression could be another target of N signaling. However, other hypotheses are also possible. For example, Rap1 has been proposed to maintain adhesion contacts between eye disc cells and therefore to facilitate cell–cell signaling. If RapGap1 is a target of Da, and Rap1 activity required for proper N and Sev signaling, this could also explain why all three pathways are reduced in *emc* mutant cells, and why restoring Rap1 activity is sufficient to rescue R7 specification. Recently, Rap1 was reported to modulate Notch signaling during eye development (Yost et al., 2023). The preferred E box binding sequence for homodimers of Da and of mammalian E proteins, CACCTG, is short and found at many locations through the genome, so it would not be surprising if unrestrained Da homodimers affected multiple gene targets (Massari and Murre, 2000; Wang and Baker, 2018). A recent study suggests that multiple da-dependent effects of *emc* mutations are due to elevated expression of the Notch ligand Delta, although it is not known whether this occurs transcriptionally (Nair and Baker, 2023 preprint).

The main conclusion from this study is that restraining the ubiquitous E protein Da can make specification of even cells that do not normally depend on proneural genes dependent on *emc*. This is in addition to the well-known role of Emc (and Id proteins) regulating processes that *do* depend on proneural proteins and/or E proteins (Bhattacharya and Baker, 2011; Ling et al., 2014; Troost et al., 2015; Wang and Baker, 2015a). The fate specification examples shown here provide still more examples of an Id protein affecting seemingly bHLH-independent processes by restraining E proteins from novel activity, not through HLH-independent function of the Id protein. This could be a common theme, given the widespread expression of E proteins.

Many molecular features of the Emc/Da system in *Drosophila* seem to be shared by mammalian Id and E protein genes, including the roles of Id proteins in E protein gene regulation (Richter et al., 2012; Schmitz et al., 2012), and stabilization of Id proteins in heterodimers (Li and Baker, 2018; Wojnarowicz et al., 2019). Notably, the effect of Id2 loss during mammalian NK cell specification is caused by E2a activity, analogous to the role of *emc* antagonizing *da* in *Drosophila* (Boos et al., 2007)*.* Indeed, the mammalian Id1 protein was originally discovered as an inhibitor of E protein function in the mammalian immune system, independent of any proneural-like bHLH proteins, although this is a case where E proteins are required in the wild type. Our results solidify the conclusion that Id proteins have two main modes of action. One is as competitive inhibitors of Ac, Sc, MyoD and perhaps some other bHLH transcription factors with master regulatory roles in specific cell fate decisions. Another is as barriers to transcriptional activation by the E proteins, represented in *Drosophila* by Daughterless, which are expressed ubiquitously and capable of interfering with many processes when unrestrained.

## MATERIALS AND METHODS

### Mosaic induction

Mosaic clones were obtained by FLP/FRT-mediated mitotic recombination technique (Golic, 1991; Newsome et al., 2000; Xu and Rubin, 1993). For non-Minute genotypes, larvae were subjected to 1 h heat shock at 37°C at 60±12 h after egg laying and for Minute genotypes, larvae were subjected to heat shock at 84±12 h after egg laying. Larvae were usually dissected ∼72 h after heat shock. Flies were maintained at 25°C.

### *Drosophila* strains

The following *Drosophila* strains were used: *emc^AP6^* (Ellis, 1994); *da^10^* (Caudy et al., 1988); [UbiGFP] M(3)67C FRT80 (Janody et al., 2004); [Ubi-GFP] FRT40; FRT82 [tub-Gal80] (Lee and Luo, 1999); P{GawB} P{GawB}lz^Gal4^ (Crew et al., 1997); *UAS- emc ^RNAi^* (VDRC KK100587) (Dietzl et al., 2007); P{TRiP.JF01766}attP2 (UAS-Ras1Gap RNAi) (Perkins et al., 2015); *rapgap1 ^22^* (Chen et al., 1997); *UAS-da-da* (Wang and Baker, 2018); and *UASpGFP-Rap1Q63E* (Ellis et al., 2013). Detailed genotypes are described in the figure legends.

### Immunohistochemistry

Antibody labeling of eye discs was performed as described previously (Bhattacharya and Baker, 2009; Firth et al., 2006). Images were recorded using BioRad Radiance 2000, Leica SP2 or SP8 Confocal microscope and processed using NIH ImageJ, and Adobe Photoshop software. Primary antibodies used were: mouse anti-Pros 1/25 (MR1A) (Spana and Doe, 1995), rat anti-Elav 1/50 (7E8A10) (Gaul et al., 1992), mouse anti-Cut 1/20 (Schroeder et al., 2011), mouse anti-Da (1:200) (Cronmiller and Cummings, 1993), guinea pig anti-Runt 1/1500 (Duffy et al., 1991), guinea pig anti-Sens 1/500 (Nolo et al., 2000), anti-E(spl) (mAb323) 1/1 (Jennings et al., 1994), mouse anti-Svp 1/1000 (Kanai et al., 2005), anti- Sevenless (Tomlinson et al., 1987), anti-RapGap1 (m4G5H3) 1/4 (Chen et al., 1997), and anti-GFP 1/500 (Invitrogen), Secondary antibodies used were multilabeling antibodies from Jackson ImmunoResearch Laboratories. To quantify RapGap1 levels, pixel intensity from maximum projections was measured in Fiji. Regions posterior to the SMW (estimated from the ELAV pattern) were compared after normalizing to control discs labelled in parallel.

Sample sizes were determined based on prior experience in similar experiments. Experiments were not blinded. No data were excluded unless parallel wild-type controls indicated technical failure.

## Supplementary Material

10.1242/biolopen.060124_sup1 Supplementary information
