# Supplementary material for: The Id protein Extramacrochaetae restrains the E protein Daughterless to regulate Notch, Rap1, and Sevenless within the R7 equivalence group of the Drosophila eye
Source: Biol Open. 2024 Aug 20;13(8):bio060124. doi: 10.1242/bio.060124 (PMC11360143; doi:10.1242/bio.060124)
Supplement: Supplementary information [file biolopen-13-060124-s1.pdf]

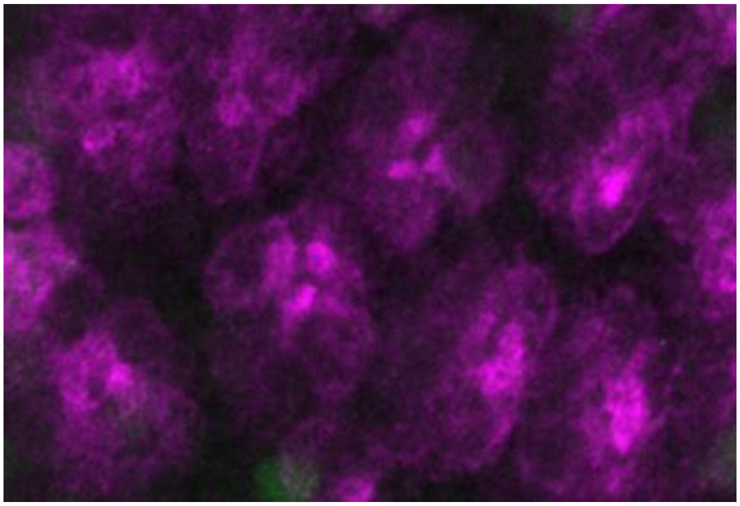

Fig. S1. For Fig. 7. Emc regulates RapGap1 levels

Enlarged portion of the eye disc shown in Figure 7C, centered on the uppermost *emc* mutant clones. RapGap1 protein in magenta, GFP in green (largely absent). RapGap1 protein is elevated generally in most or all emc mutant photoreceptor cells.

Biology Open • Supplementary information

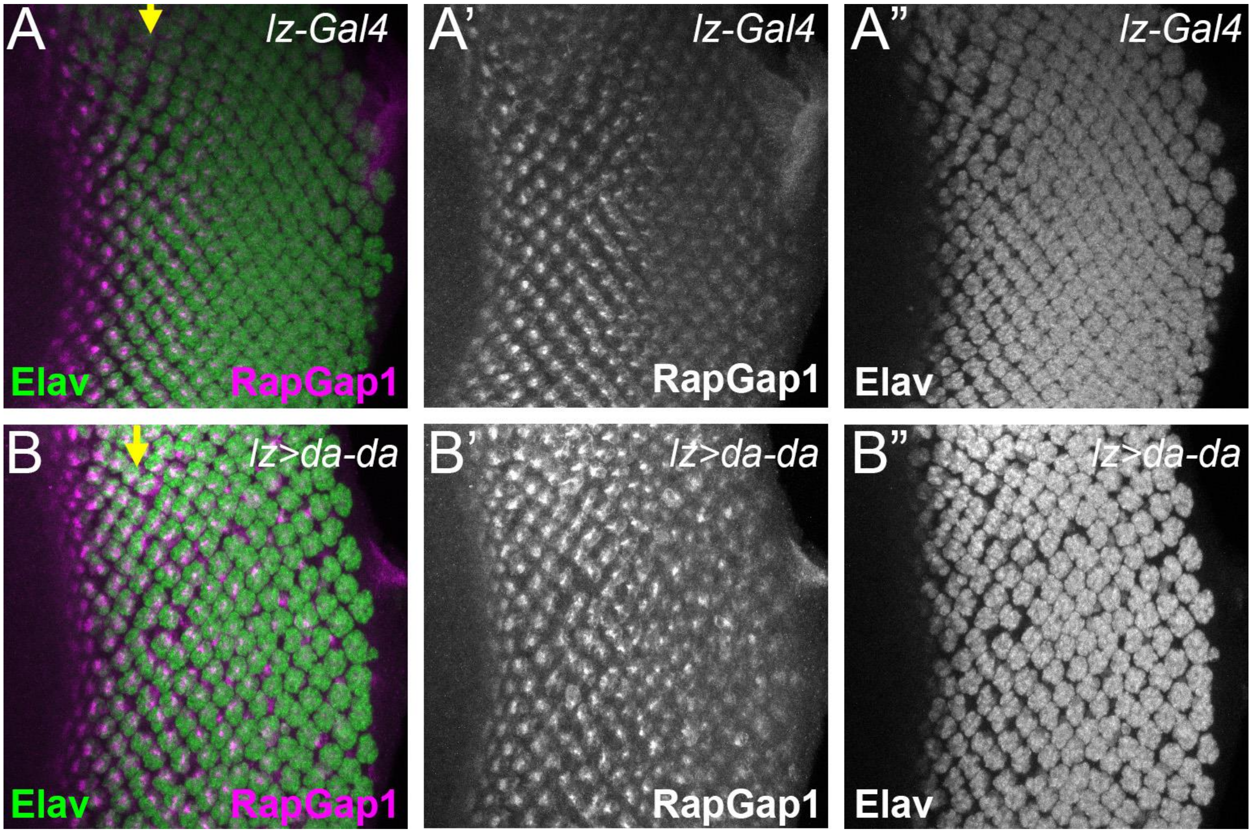

Fig. S2. For Fig. 9. Da elevates RapGap1 expression

Antibody-stained eye discs are shown. (A) In control discs RapGap1 protein (magenta) largely overlaps the photoreceptor-specific marker Elav (green). *Lz* expression begins after the Second Mitotic Wave (SMW)(Crew et al., 1997). As SMW cell divisions peak in ommatidial columns 3-5 (Baker, 2001), RapGap1 expression was measured from ~ ommatidial column 7 posteriorly (yellow arrow). (A', A") separate RaGap1 and Elav channels. N= 4. (B) Tethered Da dimer expression elevates RapGap1 expression and perturbs ommatidial patterning. Yellow arrow indicates ommatidial column 7. (B', B") separate RaGap1 and Elav channels. N=5

Genotypes: (A,A',A") *yw*, *lz-Gal4*, UAS-*GFP/+*; TM6B/+ (B,B',B") *yw*, *lz-Gal4,* UAS-*GFP/+*; UAS-*da-da*/+ (C-I) *ywhsF*; *RapGap1*^{22}; *emcAP6* FRT80/*[UbiGFP] M (3)67C* FRT80.

Biology Open • Supplementary information
